# Glutathione S-Transferase P1 Genetic Variant’s Influence on the HbA1c Level in Type Two Diabetic Patients

**DOI:** 10.3390/ijerph20021520

**Published:** 2023-01-13

**Authors:** Katarzyna Orlewska, Justyna Klusek, Stanisław Głuszek, Jolanta Klusek, Bartosz Witczak, Monika Wawszczak, Łukasz Madej, Michał Tomasz Marzec, Ewa Orlewska

**Affiliations:** 1 Collegium Medicum, Jan Kochanowski University, 25-516 Kielce, Poland; 2 Department of General, Oncological and Endocrinological Surgery, Voivodeship Hospital, 25-736 Kielce, Poland; 3 Institute of Biology, Jan Kochanowski University, 25-406 Kielce, Poland; 4 Department of Biomedical Sciences, University of Copenhagen, 1017 Copenhagen, Denmark

**Keywords:** diabetes, glutathione S-transferase, GSTP1, HbA1c

## Abstract

GST (glutathione S-transferases) are capable of influencing glucose homeostasis, probably through regulation of the response to oxidant stress. The aim of our study was to investigate the relationship between GSTP1 gene polymorphism and glycated hemoglobin (HbA1c) levels in type two diabetic (T2D) patients. A total of 307 T2D patients were included. Analysis of the GSTP1 gene polymorphism (rs1695) was conducted using the TaqMan qPCR method endpoint genotyping. HbA1c was determined using a COBAS 6000 autoanalyzer. A univariable linear regression and multivariable linear regression model were used to investigate the association between mean HbA1c level and GSTP1 gene polymorphism, age at T2D diagnosis, T2D duration, therapy with insulin, gender, BMI, smoking status. GSTP1 Val/Val genotype, age at T2D diagnosis, T2D duration and therapy with insulin were statistically significant contributors to HbA1c levels (*p* < 0.05). Multivariable regression analysis revealed that GSTP1 (Val/Val vs. Ile/Ile) was associated with higher HbA1c even after adjustment for variables that showed a statistically significant relationship with HbA1c in univariable analyses (*p* = 0.024). The results suggest that GSTP polymorphism may be one of the risk factors for higher HbA1c in T2D patients. Our study is limited by the relatively small sample size, cross-sectional design, and lack of inclusion of other oxidative stress-related genetic variants.

## 1. Introduction

Type two diabetes (T2D) is caused by defects in both insulin secretion and insulin sensitivity that result in glucose intolerance, increased gluconeogenesis, and hyperglycemia with severe complications [1]. The genetic factors that promote inappropriate homeostatic control in diabetes remain inadequately understood. Numerous studies indicate that polymorphisms in the human glutathione S-transferases (GSTs) genes leading to altered GST activity are associated with increased risk of T2D [2,3,4], yet the modulating role of GSTs in this pathway is not fully known. Early evidence demonstrated that GST functions extend beyond the enzymatic detoxification of electrophilic metabolites and xenobiotics, and some members of this family of enzymes, especially glutathione S-transferase Pi 1 (GSTP1), have been described as critical components of the redox sensing and signaling platform of the cell [5]. Given its role in stress kinase regulation, GSTP1 is unquestionably capable of influencing glucose homeostasis, probably through JNK activation [6].

GSTP1 is highly polymorphic in humans. GSTP1 gene polymorphism is most often a point mutation SNP (single nucleotide polymorphism) at exon 5, codon 313 in the GSTP1 gene, chromosome 11q13, which leads to the Ile105Val amino acid substitution. The results of mutation are GSTP1 genotypes Ile/Ile, Ile/Val and Val/Val. The exchange of isoleucine and valine in the amino acid chain results in decreased enzymatic activity of protein [7]. The Val105 variant of the enzyme may be 2–3 times less stable than the Ile105 variant and have a reduced ability to conjugate electrophilic species with reduced glutathione, leading to lower enzyme activity towards normal GSTP1 substrates, and might therefore sensitize cells to free-radical-mediated toxicity [8]. GSTP1 polymorphism may be an important factor in differential susceptibility of individuals to the toxic effects of acrolein [9]. Acrolein plays a significant role in the pathogenesis of systemic disorders such as neurodegenerative diseases [10], cardiovascular diseases [11], and diabetes [12]. Urinary acrolein correlated with glycated hemoglobin (HbA1c) in T2D patients [12] and with insulin resistance in individuals not taking insulin or oral hypoglycemic agents [13]. Thus, alterations in acrolein metabolism and detoxification due to GSTP1 gene polymorphism may potentially contribute to the difficulty in treating and maintaining desired HbA1c values among T2D patients.

The aim of the study was to evaluate the influence of genetic polymorphisms of the GSTP1 on HbA1c levels in T2D patients. HbA1c depicts mean blood glucose levels over the previous 2–3 months and is a commonly used marker of glycemic control among diabetes patients [14]. Identification of the correlation between HbA1c and diabetic complications has made HbA1c one of the most clinically useful biomarkers. A better understanding of the factors that determine glycemic control is critical to improved management of patients with diabetes mellitus.

## 2. Results Design and Methods

### 2.1. Study Population

The study group consisted of 307 unrelated patients with T2D, recruited from the diabetic outpatient center in Kielce, Poland, between 2020 and 2021. Patients were enrolled following the inclusion criteria: diagnosed T2D and aged over 18 years old, and the exclusion criteria: history of endocrine disorders, malignancies, blood disorders, liver failure, alcoholism, Cushing’s disease with treatment that can induce hyperglycemia, T1D, diabetes secondary to chronic pancreatitis, and pregnant and lactating women. The T2D diagnosis was determined by a diabetologist according to the revised criteria of the American Association of Diabetology [14]. All patients were on oral hypoglycemic agents, insulin therapy, or combination therapy. The study protocol was approved by the Ethics Committee of the Jan Kochanowski University in Kielce and all procedures were conducted according to the principles of the Helsinki Declaration. All subjects signed a written consent form for blood collection, biochemical and genetic analysis, and for using their results in this report.

Basic characteristics of participants were collected using a questionnaire including information about sex, age, age at T2D diagnosis, duration of T2D, type of administered hypoglycemic treatment, and smoking status. A current smoker was defined as a subject who continued to smoke cigarettes regularly. Obesity was defined as body mass index (BMI) ≥ 30 kg/m^2^. Two venous blood samples were collected from each patient after an overnight fast: 5 mL on EDTA tubes for molecular and HbA1c analysis, and 2 mL on NaF/EDTA for glucose analysis. HbA1c and fasting glucose were determined using a COBAS 6000 autoanalyzer, applying ROCHE methods and reagents. Samples used for DNA extraction were frozen at −20 °C until the time of genetic testing.

### 2.2. Genotyping

Peripheral blood leukocytes were the material for genetic testing. The genomic DNA was extracted from blood samples using the automatic nucleic acid extractor and genomic DNA whole blood kit (Magcore^®^, RBC BioScience, New Taipei City, Taiwan). The purity and concentration of the isolated DNA were evaluated spectrophotometrically at 260 nm and 280 nm (Denovix, DS-11). Analysis of the SNP (rs1695) polymorphism of the *GSTP1* gene was conducted using the TaqMan qPCR method—endpoint genotyping (Assay ID C_3237198_20). In all cases, the Rotor-Gene instrument by Qiagen was used. PCR amplification using ≈ 10 ng of genomic DNA was performed with an initial step of 95 °C for 10 min followed by 50 cycles of 95 °C for 15 s (denaturation step) and 60 °C for 90 s (annealing and elongation step).

### 2.3. Statistical Analysis

With the predetermined test power equal to 80% and an alpha error probability of 0.05, a minimum of 64 patients in each group (Ile/Ile, Ile/Val and Val/Val) was required to detect a clinically significant HbA1c difference of 0.5% (if this difference actually exists).

Quantitative data are described by means, standard deviations, medians, quartiles and range (minimum and maximum). Categorical data were summarized by frequencies and percentages. Genotype frequency distribution was tested against the Hardy–Weinberg equilibrium with chi-square goodness-of-fit test. Normality was checked using the Shapiro–Wilk test.

Group comparisons according to GSTP1 polymorphism were performed using the chi-square or Fisher exact test for categorical variables, and the Kruskal–Wallis test for quantitative variables (all these variables were non-normally distributed). Statistical tests were two-tailed and a *p*-value of less than 0.05 was considered significant. A univariable linear regression was conducted to investigate the association between HbA1c levels and GSTP1 gene polymorphism, as well as age at T2D diagnosis, T2D duration, gender, BMI, smoking status, and insulin therapy. Only those variables that showed a statistically significant relationship with the level of HbA1c in univariable analyses were included in the multivariable linear regression model. All statistical analyses were performed using R (version 4.0.3; The R Foundation for Statistical Computing, Vienna, Austria).

## 3. Results

A total of 307 subjects were included in our cross-sectional study, 54.7% of which were men. The majority of patients were diagnosed with T2D for more than 10 years, more than half (59%) were on insulin therapy, and 50% were classified as obese. The prevalence of poor glycemic control (defined as HbA1c > 7%) was 44.3%. A total of 130 patients were carriers of the GSTP1 Ile/Ile genotype (group A), 147 patients were carriers of the GSTP1 Ile/Val genotype (group B), and 30 patients were carriers of the GSTP1 Val/Val genotype (group C). The GSTP1 genotype distribution of participants did not deviate from the Hardy–Weinberg equilibrium (*p* = 0.2497). The characteristics of three groups of patients are presented in Table 1.

Patients in three groups were not statistically different in terms of age and sex distribution as well as T2D duration, age at T2D diagnosis, BMI, smoking status, insulin therapy and fasting blood glucose level. We observed statistically significant effects of GSTP1 polymorphism on mean HbA1c level and percentage of patients with HbA1c > 7% (Table 1). The highest mean HbA1c values (7.6; SD 1.3) and the highest percentage of patients with HbA1c > 7% (63.3%) were seen in carriers of the GSTP1 Val/Val genotype (group C).

Univariable regression analysis showed that not only the GSTP1 Val/Val genotype, but also age at T2D diagnosis, T2D duration and insulin therapy were independent variables that were identified as statistically significant contributors to HbA1c levels (*p* < 0.05) (Table 2). Multivariable regression analysis revealed that GSTP1 (Val/Val vs. Ile/Ile) is associated with higher HbA1c levels even after adjustment for variables that showed a statistically significant relationship with the level of HbA1c in univariable analyses (Table 2). The linear regression multivariable model explained 24.8% of the variance in HbA1c levels (adjusted r-squared = 0.248, F (5,301) = 21.27, *p* < 0.0001).

## 4. Discussion

In this study we investigated the relationship between GSTP1 gene polymorphism and HbA1c levels in Polish T2D patients. We found that HbA1c levels were significantly higher in carriers of GSTP1 105Val/Val mutation than in patients with the wild genotype GSTP1 105Ile/Ile. Regression modelling suggests that other factors such as earlier age of T2D diagnosis, longer duration of T2D and insulin therapy were each found to positively correlate with HbA1c levels. GSTP1 (Val/Val vs. Ile/Ile) was associated with higher HbA1c values even after adjustment for variables that were identified as significant contributors to HbA1c levels (*p* < 0.05). Previous study also observed that age, age of T2D diagnosis, T2D duration and insulin therapy were associated with HbA1c [15,16], but the effects of GSTP1 polymorphism on HbA1c reported in our study is novel.

Given its role in cellular detoxification, maintenance of cellular redox homeostasis and stress kinase regulation, GSTP is capable of influencing glucose homeostasis, probably through regulation of the response to oxidant stress, generation, and adduction of critical proteins or JNK (c-Jun N-terminal kinase) activation [6,9]. Evidence suggests that the polymeric forms of GST proteins, arising from single-nucleotide polymorphisms (SNPs), have altered enzyme activity [17]. It was shown that the presence of valine at position 105, which is part of the H-site, disrupts the water hydrogen-bonding network, allowing GSTP to accommodate less bulky substrates [18]. The presence of valine at position 105 yields lower enzyme activity toward acrolein [9], which may contribute to insulin resistance and higher HbA1c values through the oxidative and inflammatory toxicities of acrolein [12,13,19,20]. Polymorphic forms of human GSTP1 differ in their ability to regulate Prdx6 peroxidase function, a feature that may influence human population susceptibilities to oxidant stress [21]. It has been reported that the variant of the GSTP1-Val genotype might contribute to declined antioxidant activity in patients with heart failure and cardiovascular diseases [22,23,24,25,26]. If these results are translated to the setting of our study, it is speculated that GSTP1∗Val/Val carriers might possibly have a lower antioxidant potential providing a worse environment for glycemic control. Picu et al. [27] has shown a positive correlation between total oxidant status and HbA1c. Khosrowbeygi et al. [28] observed significant negative correlation between values of HbA1c and total antioxidant capacity. This highlights that oxidative stress is a potential covariate in predicting patient response to antidiabetic treatment, and GSTP1 genotyping can be relevant for better identification of T2D patients who are likely to need specific pharmacological strategies focusing not only on lowering glucose [29], but also on oxidative stress. Many studies showed that Val allele of the *GSTP1* Ile105Val polymorphism and the *GSTP1* Val/Val genotype play an important role in individual susceptibility to T2D in different populations [4,30,31,32,33,34,35], but only one study investigated the relationship between GSTP1 gene polymorphism and HbA1c level in T2D patients [31]. This study demonstrated no effect of polymorphism in the GSTP1 gene on glycemic control parameters, but in the population included in this study (n = 300) the GSTP1105Val/Val genotype, which significantly influenced the level of HbA1C in the Polish diabetic population, has not been detected. It was found that the mean HbA1c levels in carriers of the Ile/Ile genotype and Ile/Val genotype were not significantly different, which was also observed in our study. Taken together, our findings are the first to identify the potential association of the GSTP1 Val/Val gene variant with HbA1c levels in the Polish T2D population. Testing the association of GSTP1 gene variants with HbA1c and considering other genetic and non-genetic factors could help to determine which T2D patients will respond well to specific treatments, and to identify molecular targets for future individualized therapy. To date, the relationship between glycemic control and oxidative stress has received limited attention in clinical studies, but recently published randomized controlled trials demonstrated that supplementation with substances with anti-oxidative properties, e.g., delta-tocotrienol or resveratrol, in addition to hypoglycemic agents, improved glycemic control in T2D patients [36,37]

## 5. Limitations

Our study is limited by the cross-sectional design and the relatively small sample size. Based on sample size calculation with an alpha error probability of 0.05 and test power of 0.8, a minimum of 64 patients in each group (Ile/Ile, Ile/Val and Val/Val) was required to detect a clinically significant HbA1c difference of 0.5% (if this difference actually exists). In our study the sample size in the Ile/Ile, Ile/Val groups exceeded the minimum value, but the Val/Val group consists of only 30 patients (9.8% of 307). The problem, however, is not that the sample size is too small because the difference between Ile/Ile and Val/Val has been shown and it was on the level 0.6%, which is slightly above the minimum clinically significant difference of 0.5%. No difference in relation to HbA1c was shown between Ile/Ile and Ile/Val (the difference here was 0.045%), because this difference probably does not exist (and the samples here are much more numerous than assumed: n = 64). The problem of insufficient sample size would be important if the difference observed for the collected data between Ile/Ile and Ile/Val exceeded 0.5, and *p* in the calculations would be >0.05 but such a phenomenon did not occur.

In the case of T2D, where both genetic and environmental components are important throughout the duration of the disease, it is difficult to show the unambiguous influence of individual factors. We cannot entirely rule out confounding factors, although regression analysis has been used to minimize the impact of non-genetic factors (such as age, gender, BMI, smoking, disease duration) on the results of our study.

In order to limit the differentiation of external determinants, recruitment was carried out in a single center. This ensures uniform standards of treatment and care (equal access to diagnostic tests, modern methods of treatment, specialist consultations, dietitian advice) for T2D patients and our study group. Moreover, the study group comes from one geographical region and is ethnically homogeneous.

We are aware that only one SNP within GSTP1 is not enough to elucidate the role of this gene in the glucose homeostasis. Genome-wide association studies in the future should be conducted to investigate the association between other SNPs in the *GSTP1* gene and glycemic control parameters in T2D patients. Future studies should also be performed to see if the *GSTP1* Ile105Val polymorphism or other SNPs in this gene are causally triggering higher HbA1c levels through mediating the expression of this gene in specific tissues, e.g., pancreas, liver, muscles.

## 6. Conclusions

It may be concluded that the GSTP1 Val/Val genotype is significantly associated with higher HbA1c levels in Polish T2D patients, regardless of the other factors such as age at T2D diagnosis, T2D duration, insulin therapy, gender, BMI, and smoking status. The potential effect of GSTP1 polymorphism on this parameter of glycemic control, most probably through regulation of response to oxidant stress, might be useful in better selection of T2D patients who need specific pharmacological strategies focusing not only on lowering glucose, but also on oxidative stress.

## Figures and Tables

**Table 1:** Demographic, clinical, and biochemical characteristics of the study population.

Variables	Group A Ile/Ile (N = 130)	Group B Ile/Val (N = 147)	Group C Val/Val (N = 30)	*p*-Value (for A, B, C Group Comparison)
Age				0.9949
Mean (SD)	67.2 (8.4)	67.2 (7.6)	67.4 (5.6)	
Median (Q1, Q3)	67.0 (62.2, 73.0)	67.0 (63.0, 72.0)	67.0 (63.2, 71.0)	
Range	47.0–90.0	47.0–82.0	54.0–79.0	
Sex				0.203
Women	62 (47.7%)	68 (46.3%)	9 (30.0%)	
Men	68 (52.3%)	79 (53.7%)	21 (70.0%)	
Age at diabetes diagnosis (in years)				0.4376
Mean (SD)	54.3 (10.3)	55.1 (9.6)	52.5 (10.3)	
Median (Q1, Q3)	54.5 (48.0, 60.0)	56.0 (51.0, 61.0)	54.0 (46.5, 59.8)	
Range	26.0–88.0	27.0–76.0	31.0–69.0	
Diabetes duration (in years)				0.3598
Mean (SD)	12.9 (7.8)	12.2 (7.7)	14.9 (10.0)	
Median (Q1, Q3)	12.0 (7.0, 17.0)	10.0 (6.0, 15.0)	11.5 (7.5, 20.0)	
Range	1.0–35.0	2.0–40.0	2.0–39.0	
BMI				0.2219
Mean (SD)	30.9 (5.0)	30.5 (4.2)	29.3 (4.8)	
Median (Q1, Q3)	30.8 (27.1, 34.1)	30.1 (28.1, 32.6)	28.5 (26.4, 31.9)	
Range	22.3–47.9	19.2–43.4	19.6–40.6	
Smoking				0.8169
No	112 (86.2%)	130 (88.4%)	27 (90.0%)	
Yes	18 (13.8%)	17 (11.6%)	3 (10.0%)	
Insulin therapy				0.3879
No	53 (40.8%)	64 (43.5%)	9 (30%)	
Yes	77 (59.2%)	83 (56.5%)	21 (70%)	
Fasting glucose				0.8134
Mean (SD)	120.7 (22.2)	122.4 (23.0)	124.7 (26.2)	
Median (Q1, Q3)	116.0 (107.0, 133.0)	119.0 (108.0, 129.5)	118.0 (109.5, 138.8)	
Range	80.0–185.0	78.0–236.0	91.0–226.0	
HbA1c				0.0235
Mean (SD)	7.0 (1.1)	7.0 (1.2)	7.6 (1.3)	
Median (Q1, Q3)	6.9 (6.1, 7.7)	6.7 (6.2, 7.6)	7.3 (6.8, 8.1)	
Range	5.0–10.5	4.3–11.7	5.3–11.0	
HbA1c > 7%				0.0293
No	69 (53.1%)	91 (61.9%)	11 (36.7%)	
Yes	61 (46.9%)	56 (38.1%)	19 (63.3%)	

**Table 2:** Results of univariable and multivariable regression analyses with HbA1c level as the outcome variable.

	Univariable Analysis			Multivariable Analysis		
Variable	*Beta*	95% CI	*p*-Value	*Beta*	95% CI	*p*-Value
Age at diabetes diagnosis (years)	−0.037	(−0.050)–(−0.025)	<0.0001	−0.026	(−0.042)–(−0.009)	0.002
Diabetes duration (years)	0.043	0.027–0.059	<0.0001	0.021	0.00001–0.041	0.0499
Gender (reference level = female)	0.042	(−0.225)–0.309	0.756	-	-	-
BMI (kg/m^2^)	0.014	(−0.014)–0.043	0.325	-	-	-
Smoking (reference level = no)	−0.134	(−0.538)–0.269	0.512	-	-	-
GSTP1 wild type (Ile/Ile) Ile/Val Val/Val	Reference level −0.045 0.599	(−0.323)–0.232 0.132–1.065	0.745 0.012	−0.011 0.510	(−0.274)–0.252 0.042–0.953	0.932 0.024
Insulin therapy (reference level = no)	1.149	0.912–1.387	<0.0001	1.005	0.735–1.276	<0.0001

## Data Availability

All data are available on reasonable request.
